# Integrating implementation science and intervention optimization

**DOI:** 10.1186/s13012-025-01457-0

**Published:** 2025-10-03

**Authors:** Kate Guastaferro, Corrina Moucheraud, Jonathan Purtle, Linda M. Collins, Donna Shelley

**Affiliations:** School of Global Public Health, New York University, 708 Broadway, Room 636, New York, NY 10003 USA

**Keywords:** Intervention optimization, Multiphase optimization strategy (MOST), Factorial experiment

## Abstract

**Background:**

Implementation scientists increasingly recognize the value of multiple strategies to improve the adoption, fidelity, and scale up of an evidence-based intervention (EBI). However, with this recognition comes the need for alternative and innovative methods to ensure that the package of implementation strategies work well within constraints imposed by the need for affordability, scalability, and/or efficiency. The aim of this article is to illustrate that this can be accomplished by integrating principles of intervention optimization into implementation science.

**Method:**

We use a hypothetical example to illustrate the application of the multiphase optimization strategy (MOST) to develop and optimize a package of implementation strategies designed to improve clinic-level adoption of an EBI for smoking cessation.

**Results:**

We describe the steps an investigative team would take using MOST for an implementation science study. For each of the three phases of MOST (preparation, optimization, and evaluation), we describe the selection, optimization, and evaluation of four candidate implementation strategies (e.g., training, treatment guide, workflow redesign, and supervision). We provide practical considerations and discuss key methodological points.

**Conclusion:**

Our intention in this methodological article is to inspire implementation scientists to integrate principles of intervention optimization in their studies, and to encourage the continued advancement of this integration.

Contributions to the literature

- Intervention optimization has been applied in the development of multicomponent behavioral interventions that target a broad array of health outcomes.
- The public health impact of any behavioral intervention—even one that has been optimized – is likely to be hindered by implementation challenges.
- This paper demonstrates how this rigorous framework of intervention development and optimization may be used to develop an optimized, theory-driven multicomponent implementation strategy.

## Background

A consensus is emerging that although billions of dollars have been invested in development of behavioral, biobehavioral, biomedical, and social-structural interventions (hereafter, behavioral interventions), these interventions are falling short of achieving their intended public health impact [28], where impact is operationalized as the product of effectiveness and reach [23]. The science supporting the implementation of interventions is ripe for an innovative approach [16, 19]. Indeed, for the past two decades, there have been calls for approaches to accelerate the research-to-practice translation pipeline [7, 17, 22, 26, 35, 60]. The field of implementation science emerged as one answer to this call [19], with a major focus on developing empirically-supported strategies, methods or actions that facilitate the adoption, implementation, sustainability, and scale up of an evidence-based intervention (EBI) in health care and public health settings [1, 2, 14, 31, 40, 44, 47, 48, 63]. Implementation scientists have developed taxonomies for defining distinct strategies, and categories of strategies [31, 45–47].

In practice, multiple strategies (e.g., bundles, sets, packages) are selected (and often needed) to ensure the successful implementation of an EBI. While we may have compendia of candidate implementation strategies to facilitate the adoption, implementation, sustainability and scale up of EBIs, the continued testing of packages of implementation strategies remains a barrier to true progress. For example, a package of implementation strategies could include: building a coalition, and conducting ongoing training, and introducing reminders (for a comprehensive list of implementation strategies see for example [46]. A package of implementation strategies is most frequently evaluated in a two-arm randomized controlled trial (RCT) design with the purpose of testing the strategies' effect on implementation outcomes and/or EBI effectiveness. Despite being viewed as the gold standard for developing evidence, the two-arm RCT design in this case precludes the field from developing a robust evidence of the mechanisms by which implementation strategies are most effective, the individual contributions of those strategies, and whether (and how) strategies might interact either synergistically or antagonistically to influence outcomes [45, 46].

In the last 20 years [9], a new research paradigm, intervention optimization, has emerged as a solution to this shortfall in behavioral interventions (i.e., EBIs). An optimized intervention is comprised of components that produce the best expected outcome of interest that can be obtained while also being practical to implement (readers are referred to [8] for a full treatment of the differences between the classical approach and intervention optimization). This innovation arose because the same shortfalls noted above were experienced in the development of EBIs, which often included multiple components (e.g., Quitline counseling, tailored nurse-delivered counseling,text messages sent twice per day for 8-weeks; nicotine replacement therapy [50, 51] immediately packaged together and evaluated in a two-arm RCT design. This approach precluded the development of an evidence base about which components are most effective, for whom, and under what conditions, and provides only limited information as to how the components work in the presence and absence of one another [11]. In this paper, we argue that intervention optimization has relevance also for implementation scientists interested in designing and testing packages of strategies.

For the purposes of this article, let us consider a package of implementation strategies to be a type of intervention that can be optimized [25]. Using the intervention optimization paradigm, it would be possible to empirically identify the combination of implementation strategies that produces the best expected outcome given implementation constraints imposed by the need for affordability, scalability, and efficiency. We will assume the investigator has an established and adequately evaluated EBI – in our hypothetical example, a smoking cessation intervention inspired by the work of [50, 51]. The remaining question is how to best implement the EBI in real world settings. Imagine the investigators have encountered barriers to adoption at the system-, organizational- and individual-levels. Using a hypothetical experimental design based on a real implementation science study, we demonstrate here how the principles of intervention optimization, and specifically the multiphase optimization strategy (MOST; [11]), could be integrated into implementation science.

## Overview of the intervention optimization paradigm

Intervention optimization is the process of achieving a strategic balance of effectiveness against desired qualities of affordability, scalability, and efficiency (the strategic balance is referred to as intervention *EASE*; [11, 12]. One approach to intervention optimization is MOST. Though MOST has been described in detail in multiple outlets [8, 11, 12], here we provide a brief overview of MOST with a specific focus on the implications for the field of implementation science.

Drawing on principles from the fields of behavioral science, engineering, implementation science, economics, and decision science, MOST is a principled framework to develop, optimize, and evaluate multicomponent behavioral interventions. In MOST a set of intervention components is identified, but in contrast to the classical approach, these are considered *candidate* components; they are candidates that may or may not ultimately be included in the intervention. The decision will be based on their performance in an experiment and on other information, such as cost.

MOST is composed of three phases, each with specific objectives and activities: preparation, optimization, and evaluation. In the *preparation phase*, the goal is to lay the foundation for optimization by developing a theoretically and empirically derived conceptual model, identifying candidate intervention components (here, implementation strategies), conducting pilot work, and specifying an optimization objective. A theoretically- and empirically-derived conceptual model depicts the process upon which the investigators wish to intervene –it is the blueprint for how the intervention will be built (i.e., which components) and how it will produce the desired outcome. Pilot work is focused on ascertaining the acceptability and feasibility of the components. In addition, pilot work may also be necessary to undertake pilot testing in order to refine candidate strategies to the local context and can help refine study protocols.

In the optimization phase, candidate intervention components are empirically studied in an optimization RCT. The optimization RCT serves a different purpose, and is logically different, from the standard evaluation RCT. Optimization RCTs may use any of a variety of experimental designs, but the design must be selected based on the resource management principle of MOST, which holds that an investigator must make efficient use of research resources. For this reason, typically (but not always), the experimental design selected for the optimization trial is drawn from the family of factorial experiments (e.g., fractional factorial, sequential multiple assignment randomized trial [SMART; [29, 36]), microrandomized trial (MRT; [49]). Such experimental designs enable systematic assessment of the performance of the components, independently and in combination, on the outcome (or outcomes) of interest. One common optimization RCT design is the 2^ k^ factorial experiment wherein each of the *k* factors has two levels.

Although it is beyond the scope of this article to detail the efficiency and design of factorial experiments (see [10]), we take this opportunity to make some key methodological points, and provide recommended readings for those who are interested. First, an optimization RCT (such as the factorial experiment) is a randomized, controlled experimental design. For this reason, we call it an RCT despite its differences from the evaluation RCT which is the evaluation of the optimized intervention. Second, given that intervention optimization research questions call for assessing the individual and combined performance of intervention components, a factorial experiment that has been properly designed and conducted, and analyzed in the standard manner using effect (−1,1) coding, often requires many *fewer* participants to maintain a given level of statistical power, as compared to alternative experimental designs (e.g., a two-arm evaluation RCT for each component, or a multiple-arm comparative experiment; for a detailed explanation of why, see [10]. Third, the methods required to analyze data from a factorial experiment (i.e., factorial analysis of variance) have been in existence for more than 100 years [21] and are familiar to nearly all biostatisticians. Finally, it is common for implementation trials to require cluster randomization (e.g., clinic, community, school or organization), and many optimization trial designs, including the factorial, can accommodate this need. Notably, assuming the target effect size is the same, the required sample size for a clustered, or multilevel, 2^ k^ factorial experiment would most likely not be appreciably greater than what would be required in a traditional two-arm clustered RCT.

Empirical data from the optimization RCT, along with data on implementation resource requirements (e.g., cost, time, etc.) and constraints (e.g., fixed budget, maximum number of labor hours, etc.) are used to identify the set of components and component levels that produce the best expected outcome given constraints (i.e., the optimized intervention; or if conducting an implementation science trial, the optimized implementation strategy). The ultimate selection of the optimized intervention is based on the results of the optimization RCT, the data on resource requirements and constraints, and the empirical data and the optimization objective [55], which is identified by the investigator during the preparation phase. The optimization objective specifies the way in which effectiveness will be balanced with implementation constraints imposed by the need for affordability, scalability, and/or efficiency (e.g., cost per patient, provider time to deliver intervention, etc.). Finally, in the evaluation phase, the optimized intervention may be subjected to the familiar evaluation RCT (typically a two-arm RCT) to ascertain effectiveness, with a strengthened understanding of the inner workings of the intervention, and what it would mean in practical terms to implement it.

To concretize how intervention optimization can be used by implementation scientists, we offer a hypothetical example.

### Hypothetical example: optimization of a package of implementation strategies designed to increase the adoption of a smoking cessation intervention

In this hypothetical example, a team chooses to use MOST to optimize a package of implementation strategies to improve adoption of a smoking cessation intervention (the EBI). We describe activities to be accomplished in the three phases of MOST.

### Preparation phase

As alluded to previously, implementation research has tools for identifying and defining implementation strategies (e.g., implementation mapping; [20]). In our hypothetical example, the team decides to use Consolidated Framework for Implementation Research [CFIR]; [16, 34]) to identify barriers to and facilitators of implementation of the smoking cessation EBI, and potential mediators of the implementation outcomes,and uses the CFIR – Expert Recommendations for Implementing Change (ERIC) Matching Tool to select implementation strategies that are hypothesized to address the identified barriers and facilitators to affect the needed change in provider behavior for increasing adoption of the EBI [61]. Stakeholder engagement and mixed methods formative research, guided by the above frameworks, may also be used by our hypothetical team to inform the conceptual framework, strategy selection, and design or operationalization of the strategies [50, 51].

**Fig. 1:**
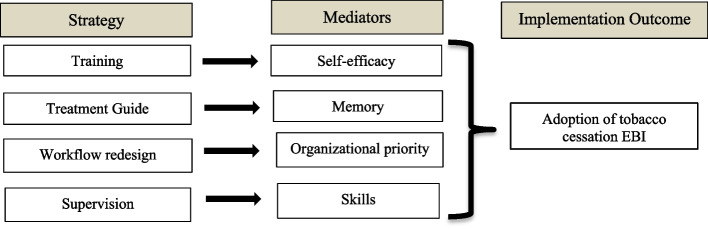
Conceptual model for the implementation strategies to increase EBI adoption

Ultimately, in this hypothetical example, four candidate implementation strategies are selected: (1) Training all health care workers with or without a booster session (designed to increase their self-efficacy to deliver the EBI); (2) A tobacco use treatment guide that outlines suggested questions and probes that health care workers can use when offering brief advice to quit (designed as a memory aid for delivering the EBI); (3) Workflow redesign that aligns new protocols with current processes and resources (designed to align organizational priorities around the EBI); and (4) Supportive supervision provided by a member of the research team with expertise in both tobacco use treatment and implementation facilitation (designed to enhance skills to deliver the EBI). Figure 1 depicts the conceptual model for this hypothetical example, and highlights the mediators (sometimes referred to as mechanisms) that each component (implementation strategy) targets.

The implementation outcome of interest is the adoption (or uptake) of the EBI [13, 48, 50, 51] and the investigators operationalize adoption as the percentage of clients who report that they received the EBI during the visit (i.e., clinician-delivered advice to quit, brief counseling and referral to treatment) during an exit interview. The identification of the strategies, determinants, and outcomes are opportunities to include input from stakeholders. The team could also engage stakeholders in discussing the relevant optimization objectives – any number of objectives could be identified by different stakeholders in different contexts. Suppose in this hypothetical example, provider time is the greatest constraint in the local context. For this reason, the selected optimization objective is to identify the intervention version that maximizes adoption of the EBI within an agreed-upon amount of maximum required provider (i.e., physician) time to execute the implementation strategies.

### Optimization phase

The research question for the optimization phase is: *what is the individual and combined effect of the components on adoption?* For this hypothetical experiment, the team chooses to conduct a 2^4^ factorial design to estimate the main effects of the four factors on the outcome of adoption: *TRAINING, TREATMENT GUIDE, WORKFLOW REDESIGN, and SUPERVISION*, and interactions involving these factors.

The optimization trial is designed as a cluster RCT adequately powered for detection of main and interaction effects where health facilities (clusters) are randomized to 1 of the 16 experimental conditions representing all possible combinations of the 4 factors, as seen in Table 1 (for more on power of a factorial experiment see [18, 37]). To examine the need for enhanced training time, the team chose to set the levels for training as “basic” (i.e., two days)” or “enhanced” (basic plus a booster training).” To explore varied doses for the supportive supervision implementation strategy, the team chooses to compare a single supervision visit to three bimonthly visits. For the treatment guide and workflow redesign strategies, the investigators choose to set the levels as “on” or “off.”

**Table 1:** Experimental conditions for 2^4^ factorial experiment

Experimental Condition	TRAINING	TREATMENT GUIDE	WORKFLOW REDESIGN	SUPERVISION	% adopted ($\widehat{Y})$
1	Basic	On	On	Single	65
2	Basic	On	On	3 visits	90
3	Basic	On	Off	Single	15
4	Basic	On	Off	3 visits	40
5	Basic	Off	On	Single	63
6	Basic	Off	On	3 visits	88
7	Basic	Off	Off	Single	13
8	Basic	Off	Off	3 visits	38
9	Enhanced	On	On	Single	75
10	Enhanced	On	On	3 visits	100
11	Enhanced	On	Off	Single	25
12	Enhanced	On	Off	3 visits	50
13	Enhanced	Off	On	Single	73
14	Enhanced	Off	On	3 visits	98
15	Enhanced	Off	Off	Single	23
16	Enhanced	Off	Off	3 visits	48

Typically, the analysis of factorial optimization RCT data uses factorial analysis of variance (ANOVA) with effect coding (i.e., + 1, −1). We assume a perfectly balanced factorial experiment in which main and interaction effect terms are uncorrelated, and for this reason the team is sufficiently powered to estimate up to the *k*-way interaction [18, 37]. Because this is a hypothetical example, ANOVA results are not displayed. Table 1 displays the hypothetical expected adoption outcome and a hypothetical calculation of provider time required for each experimental condition (i.e., combination of implementation strategies). We note that it is possible to explore moderators and mediators in factorial ANOVA to better understand hypothesized effects among subgroups or contexts [56], which is key for addressing questions of “for whom and under what conditions” facets of optimization.

**Fig. 2:**
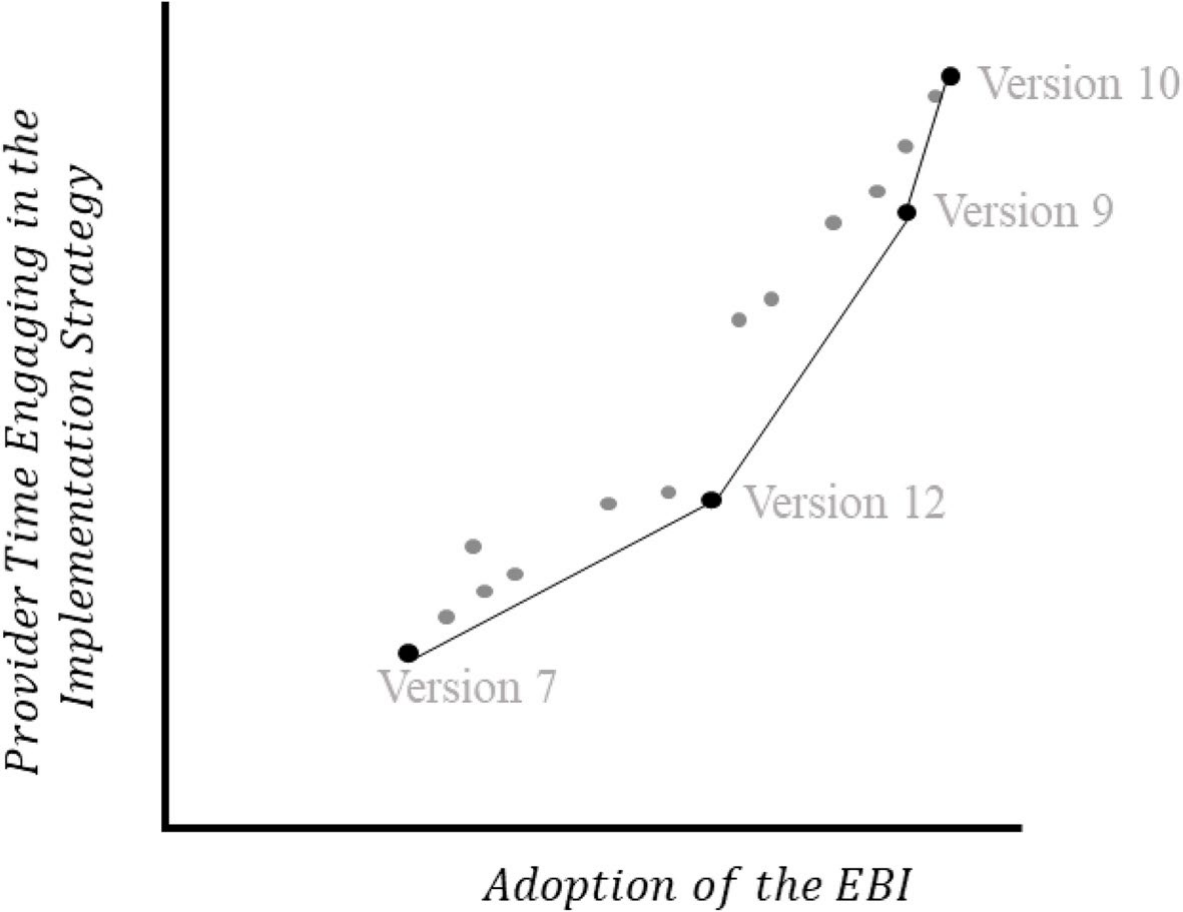
DAIVE output for a hypothetical 2^4^ factorial optimization RCT. Legend: For simplicity, we label only the versions of the intervention that fall on the frontier of efficiency with the experimental conditions defined in Table 1: Version 10 corresponds to Experimental Condition 10, which consists of Enhanced Training, Treatment Guide, Workflow Redesign, and 3 supervision visits; Version 10 corresponds to Experimental Condition 9, which consists of Enhanced Training, Treatment Guide, Workflow Redesign, and a single supervision visit; Version 12 corresponds to Experimental 12, which consists of Enhanced Training, Treatment Guide, and 3 supervision visits; and Version 7 corresponds to Experimental Condition 7, consisting of Basic Training, Workflow Redesign, and a single supervision visit

Once the results from the optimization RCT and data on cost have been obtained, it is possible to empirically select (i.e., decide on) an optimized intervention. The current suggested approach to decision-making in MOST is decision analysis for intervention value efficiency (e.g., “DAIVE”; [55, 57]). Using posterior expected values [5], DAIVE makes use of all available empirical information from the optimization RCT to support decisions in selecting an optimized intervention that maximizes expected value, in contrast to relying on arbitrary thresholds (e.g., statistical significance) to determine what effects were ‘important’ enough [57]. A plot of intervention performance in terms of expected outcomes (adoption of the EBI, *x*-axis) and resource requirements (provider time related to execution of implementation strategies, *y*-axis) is produced using DAIVE. Hypothetical DAIVE output is displayed in Fig. 2. The solid line indicates alternative interventions considered ‘value efficient’ because they produce better adoption outcomes for less provider time – for this reason, the selected optimized intervention typically falls on this line. Using the DAIVE output, investigators can visualize the balance between adoption of the EBI and provider time in service of selecting an optimized intervention. Deciding on the optimized intervention requires careful consideration of constraints (i.e., provider time) and determining what a meaningful effect of the EBI. For example, the team, with stakeholder input, might also define a maximum amount of provider time that can be spent on this activity, and focus only on those implementation strategy combinations beneath this “ceiling” on the y-axis.

### Evaluation phase

The research question for the evaluation phase is: *how does the optimized intervention compare to a suitable control*? Typically, this question is suitable for an evaluation RCT design, such as a two-arm RCT. For example, [42] conducted a two-arm evaluation RCT to compare an optimized smoking abstinence intervention to usual care. Pfammatter et al. [41] are currently conducting a non-inferiority trial to compare an optimized intervention designed to maximize weight loss to the standard of care (Diabetes Prevention Program).

In our hypothetical example, suppose the investigators select the set of implementation strategies in which clinics receive enhanced training, the training guide, the flow redesign and single session supervision (experimental condition 9). In the evaluation phase they would test this optimized intervention to a suitable control – standard of care, no intervention control, attention control – using a two-arm evaluation RCT.

However, it is important to note that the evaluation phase research question is not limited to a two-arm evaluation RCT design. The experimental design should be driven by the research question. For example, [59] conducted a hybrid evaluation-optimization RCT using a 2^2^ factorial experiment thereby testing the effectiveness of an optimized intervention designed to reduce sexually transmitted infections among first year college students (itMatters) compared to an active control while simultaneously estimating the effect of an added sexual violence prevention component which was of interest to the schools who would deliver itMatters. In this design, the team was thereby able to complete the evaluation phase for itMatters while also working towards assessing the contribution of a sexual violence prevention component. This highlights a core principle of MOST – the continual optimization principle which suggest that even an optimized intervention can be further optimized.

## Discussion

Intervention optimization has been used in hundreds of projects funded by the US federal organizations such as the National Institutes of Health and Institute of Educational Sciences, as well as being increasingly funded in Europe, Asia, and Australia [3, 6, 24, 27, 43, 54, 59, 62, 64]. However, MOST has been used minimally in the field of implementation science, with regard to either advancement of methods e.g., [58] or substantive applications. There are noteworthy exceptions, such as the optimization of the implementation of Family Navigation, an EBI designed to reduce disparities in accessing mental and behavioral services in a pediatric clinic [4], and the optimization of a package of implementation strategies designed to improve implementation of an evidence-based psychosocial program [30, 53]. This is an area of research – methodologically and substantively—ripe for innovation.

Similar to the intervention optimization paradigm, implementation science emphasizes a prospective approach to developing a theory-driven conceptual model with clearly specified hypothesized mechanisms of action that can be tested via rigorously designed trials [32, 52]. Yet, implementation studies that evaluate multicomponent strategies generally use an experimental design that precludes analyzing the mechanism of action of individual strategies and how those mediators interact to enhance or diminish implementation outcomes (i.e., two-arm evaluation RCT). Efforts to integrate intervention optimization and implementation science will be further facilitated by the use of rigorous approaches to prespecifying hypotheses about how implementation strategies work individually and in combination and a greater focus on developing validated tools to assess implementation processes and outcomes. We argue that the application of intervention optimization, particularly the use of efficient experimental designs in the optimization RCT, offers an opportunity to increase the meaningful learnings gained from these studies.

Often, implementation research is concerned with complex health interventions that target multilevel determinants of behavior [38], or addressing barriers to intervention implementation at multiple levels by selecting multicomponent and/or multilevel strategies – all generally studied in combination. For example, Perez Jolles and colleagues [39] conducted a cluster randomized trial to evaluate whether a multicomponent implementation strategy improved fidelity to the Adverse Childhood Experiences (ACE) screening and the impact of the ACE policy on child-level mental health referrals and symptom outcomes. A list of hypothesized change mechanisms was defined, but, as noted above, it was not possible, with this evaluation RCT design, to parse which of the implementation strategies (i.e., training, coaching, clinical tool and decision tools) were most effective either individually or in combination, in influencing the implementation outcomes. In contrast, Lockhart and colleagues [33] used MOST from the outset to develop a multi-level (provider and patient), multi-component intervention to increase pre-exposure prophylaxis (PrEP) prescriptions in primary care.

Intervention optimization and implementation science are further aligned in emphasizing early engagement of decision makers and other stakeholders in conversations about priorities. Both approaches use engagement processes to understand context, inform adaptation, and undertake critically important conversations about resources, priorities and other contextual factors that influence intervention or strategy selection. The goal, in both fields, is to inform theory-driven intervention or strategy development, define relevant outcomes, and explore the need for adaptations to increase implementation effectiveness in a new setting or for a new population. Integrating MOST into the pre-implementation phase of implementation research, with the specific goal of engaging stakeholders in a decision-making process about meaningful outcomes and tradeoffs between them, is an important contribution to implementation science. Similarly, using implementation science frameworks, or other approaches like implementation mapping [20], to guide the MOST preparation phase would contribute to a shared understanding of contextual factors influencing component selection and optimization decisions.

Implementation science, similar to intervention optimization, is concerned with strategically balancing outcomes with constraints, such as those imposed by the need for affordability, scalability, and efficiency [11, 12]. For example, in the hypothetical example included herein, we used the constraint of an upper limit on provider time required to engage in the implementation strategies. There are however, additional costs to consider such as the provider time to deliver the EBI, the time required of someone to train the providers to do the implementation strategies, or the cost of developing workflow redesign or providing supervision. Operationalizing constraints is specific to each study and context. In the context of implementation science research, additional outcomes may include acceptability, appropriateness, reach, and sustainability [48] which presents an opportunity for future research to consider ways in which to incorporate other implementation outcomes in the optimization objective, thereby further strengthening synergies between intervention optimization and implementation science.

We focus on the advantages of the integration of intervention optimization and implementation science, but would be remiss if we did not acknowledge more work is needed to resolve certain methodologic challenges. For example, the integration of implementation science and MOST will likely require multi-level optimization RCTs, in which different candidate components/strategies operate at different levels (e.g., health care practice, clinician, and patient). In some cases, the design of such experiments and the analysis of the resulting data will be complex. Guidelines for investigators are needed to enable selection of the most efficient and appropriate experimental designs. Another limitation is that there is currently no principled method for determining whether at the conclusion of the optimization phase the investigator should conduct an evaluation RCT; in some cases, if the results of the evaluation RCT are very strong, the investigator may even consider moving to implementation without an evaluation RCT. An approach is needed for making this important decision. There is currently much active methodological research addressing these and other topics in intervention optimization.

## Conclusion

There are myriad opportunities to merge the strengths of intervention optimization and implementation science to arrive at the ultimate goal of developing and implementing highly effective interventions that reach the intended population and attain meaningful health improvements. There are many exciting opportunities [9], for example to conduct methodological work to integrate intervention optimization into the hybrid effectiveness-implementation study framework [15],to consider best practices when applying MOST to implementation studies conducted in diverse settings including low- and middle-income countries where optimization objectives are especially salient due to resource constraints; and to optimize policy dissemination and implementation strategies. The application of intervention optimization in implementation science is an open area of science. Our intention is to encourage our colleagues to not only apply intervention optimization to their work, but also contribute to the advancement of the integration of intervention optimization and implementation science.

## Data Availability

Not applicable.
